# Regional Resilience in Times of a Pandemic Crisis: The Case of COVID‐19 in China

**DOI:** 10.1111/tesg.12447

**Published:** 2020-06-19

**Authors:** Huiwen Gong, Robert Hassink, Juntao Tan, Dacang Huang

**Affiliations:** ^1^ Department of Geography Kiel University Hermann-Rodewald-Str. 9 24098 Kiel Germany; ^2^ State Key Laboratory of Resources and Environmental Information System Institute of Geographic Science and Natural Resources Research, Chinese Academy of Sciences Beijing 100101 China; ^3^ University of Chinese Academy of Sciences Beijing 100049 China

**Keywords:** Regional resilience, economic geography, kind of shocks, pandemic crisis, COVID‐19, China

## Abstract

The notion of resilience to analyse how fast systems recover from shocks has been increasingly taken up in economic geography, in which there is a burgeoning literature on regional resilience. Regional resilience is a place‐sensitive, multi‐layered and multi‐scalar, conflict‐ridden and highly contingent process. The nature of shocks is one important impact factor on regional resilience. Arguably, so far, most literature on regional resilience has dealt with the financial crisis in 2008/2009. In this research note, we will analyse both the particular characteristics of the current COVID‐19 crisis, as well as its effects on regional recovery and potential resilience in China, where it started. We conclude that a complex combination of the characteristics of the current COVID‐19 crisis, the institutional experience of dealing with previous pandemic and epidemic crises, government support schemes, as well as regional industrial structures, might potentially affect the recovery and resilience rates of Chinese regions.

## Introduction

In current times of crisis, the notion of resilience is often used to analyse the recovery processes of systems from the shock. Resilience refers to the notion to describe that systems recover from shocks or can build up capabilities to deal with future shocks (Wilson 2018). It is a notion used in several academic disciplines, such as psychology, ecology and planning. Regional resilience is part of a broader literature on resilience in human geography, which includes urban resilience (Fastenrath *et al*. 2019), social resilience and community resilience (Wilson 2018). More recently, within human geography, economic geographers, in particular, have become interested in regional resilience in tackling the question of why some regional economies manage to renew themselves or to lock themselves out, whereas others are more locked in decline (Martin 2018; Evenhuis 2017; Lazzeroni 2019; Bristow & Healy 2020; Hassink & Gong 2020; Martin & Sunley 2020; Simonen *et al*. 2020).

The rising interest in resilience from a regional, urban and metropolitan perspective can be explained by two factors. First, the increase or perceived increase in the number of shocks and disruptions, such as natural hazards, terrorist attacks, financial crises, etc., has led to a strengthened feeling of uncertainty and insecurity. This perception is strengthened by the awareness that increasingly the modern global economy is only possible due to increasing interconnecting and interdependent global networks that are necessary but that also lead to vulnerabilities (OECD 2011). The perception is also partly caused by the influence of commercial broadcasting, internet technology and instant communication software through which people are increasingly informed about events happening in different parts of the world, which was unimaginable previously. Moreover, the financial and economic crisis in 2008–2010 has generated a boom in studies on how regional economies recovered from that crisis, and we expect a further surge during and after the current COVID‐19 crisis. Second, successful studies on socio‐ecological resilience have raised the interest in resilience from a regional, urban and metropolitan perspective. These studies were boosted by a US national research network, Building Resilient Regions, sponsored by the MacArthur Foundation between 2006 and 2013, as well as the highly citied special issue of the *Cambridge Journal of Regions, Economy and Society* published in 2010 (Christopherson *et al*. 2010). After the publication of that special issue and the critique (Hassink 2010; MacKinnon & Derickson 2013; Gong & Hassink 2017), a burgeoning conceptual and empirical literature emerged on regional resilience (for a recent bibliometric analysis, see Fröhlich & Hassink 2018).

Each crisis and shock has its own specific characteristics, but also differs concerning scale and duration, and hence has its own effects on regional economies and regional resilience (Martin 2018). So far, most literature on regional resilience has dealt with the financial crisis in 2008/2009 (see for instance Davies 2011; Sensier *et al*. 2016; Webber *et al*. 2018). In this research note, we will analyse both the particular characteristics of the current COVID‐19 crisis, as well as its interim effects on regional resilience in China, where it started, but also where lockdowns have come to an end and where we have hence a chance to give first assessments on short‐term regional resilience. Based on early observations of the current COVID‐19 crisis, we put forward three main arguments in this research note. First, the location with high shares of infected people directly correlates with regional‐economic effects of COVID‐19, with Hubei turning out to be the economically most severely hit province by the crisis, and its neighbouring provinces suffering more than other regions within China. Second, based on some first calculations of the currently available data, factors such as population density, foreign trade dependence, and the severity of the disease (infection rate per million people) have been found out to be negatively related to the short‐term economic resilience of Chinese regions. Finally, effective governmental supportive measures and regional industrial characteristics are expected to influence the long‐term economic recovery of regions hit by COVID‐19. We therefore conclude that a complex combination of the characteristics of the current COVID‐19 crisis, the institutional experience of dealing with previous pandemic and epidemic crises, government support schemes, as well as regional industrial structures, affect the potential recovery and resilience rates of Chinese regions.

In the following, we will first shortly introduce the regional resilience concept in the second section. In the third section, we will review the main literature on regional resilience during and after specific kind of shocks, namely pandemic shocks, such as COVID‐19, whereas the fourth section will give some interim assessments of the potential economic recovery of Chinese regions during and after the current COVID‐19. In the fifth section some general conclusions will be drawn.

## Regional Resilience and Different Kind of Shocks

Regional development is a shock‐prone process. (Martin 2018, p. 840)

Resilience can be considered at several scales, such as the individual, household, local, regional and national scale, and categories, such as industries, knowledge production, entrepreneurship and labour markets (Martin 2018). In addition, the disturbances take places at different scales. They range from macro‐level shocks (such as wars and financial crises) having varying effects on different places, to multi‐local shocks, for instance when a national industry collapses, to local disruption, for instance if a major plant closes (Martin 2018). Regional resilience as a conceptual framework is useful in helping us to think about regions in a dynamic, holistic and systematic way.

Two key questions that are useful for a better understanding of regional resilience are: resilient to what? And resilient of what?

The ‘to‐what‐question’ refers to the kind of disturbance, such as shocks, terrorist attacks, natural disasters, and major factory closures. Dabson *et al*. (2012) distinguish between different kind of critical events, namely *natural* events, for instance a hurricane, *human‐made* events, such as a terrorist attack or a nuclear accident, *economic* events, such as the financial crisis and the finally *medical* events, such as the current pandemic crisis.

The ‘of‐what‐question’ refers to the actors involved in a region and beyond, such as firms, workers, labour unions, policy‐makers, intermediaries, states, transnational corporations, etc. If these actors react in the same way to shocks or disturbance one can speak of collective or aggregate, generalisable reactions and mechanisms of resilience. In practice, the necessity and character of a reaction is a political question and the result of debate, negotiations and power struggles between actors inside and outside the region. Moreover, to some actors, such as regional firms, adaptation and resilience might be a success, whereas to other actors, such as international NGOs who concern more about social welfare of workers and environmental sustainability, certain types of regional resilience is viewed from a totally different perspective.

**Figure 1:**
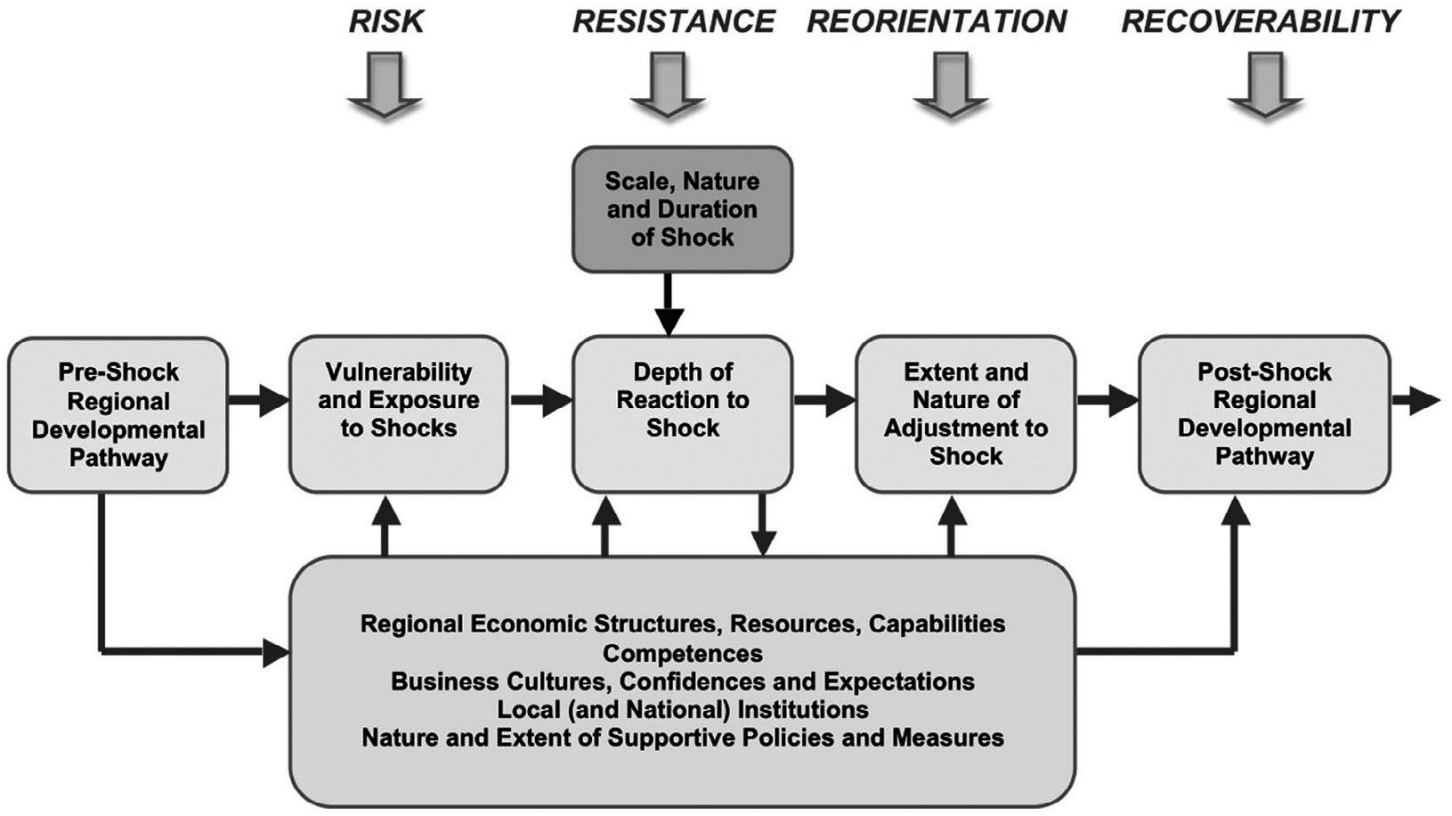
Regional resilience to recessions. *Source*: Martin et al. (2016).

Regional resilience is a process, as becomes clear in Figure 1. Before a disturbance happens, the region runs a certain degree of vulnerability, which refers to the sensitivity or propensity to be hit by a shock. The nature of the disturbance affects this risk, regions with a strong financial economy, for instance, were more vulnerable to the financial crisis in 2008/2009 than regions with a weak financial economy. Resistance refers to the depth of a reaction to a shock, which is relatively easy to measure with quantitative indicators. Reorientation, recoverability and reorganisation after a disturbance refer to adjustment and regional development pathways and are affected by structural changes that occur, and hence often require a longer period to evaluate.

The several stages of this development are influenced by four groups of impact factors we can find in regions and beyond (Figure 1). First, regional economic structures, resources, capabilities and competences, influence regional resilience in the different stages (Brown & Greenbaum 2016; Martin *et al*. 2016). Some regions are more resilient than other regions, because of the regional industries dominating the regional economy are less affected by the shocks (see example above). Second, business culture, confidence and expectations affect regional resilience (Martin & Sunley 2020), and thirdly, local and national institutions, including norms and values (Huggins & Thompson 2015) and experiences to deal with past shocks and crises (institutional learning) (Bristow & Healy 2014). Fourthly, the nature and extent of supportive policies and measures, such as specific policies to remedy the crisis, etc., at several spatial scales affect regional resilience (Cowell 2015; Evenhuis 2016; Kakderi & Tasopoulou 2017). The role of the state and relevant policy instruments at several spatial levels, in fact, are of utmost importance in analysing and explaining differences in crisis response and regional economic adaptability.

In recent conceptual work, Martin and Sunley (2020, p. 32) emphasise the interdependence between regional development and regional resilience, by stating that ‘The relationship is a recursive one: the features and structures built up by a region’s past development influence its resilience, and its resilience to shocks will impact back on that development path, either reinforcing it or promoting change’. The latter refers to transformational opportunities related to shocks and regional resilience. Moreover, they point at the relation between the intensity and duration of the shock and the different kind of transformations that could take place: ranging from low intensity and short duration, which would lead to bounce‐back resilience, to high intensity and long duration, which could potentially lead to transformative resilience (Manca *et al*. 2017; Martin & Sunley 2020).

In the current debate about regional resilience, there is disagreement about whether regional resilience should also deal with long‐term structural change and slow burn, or only with shock situations (Martin 2018; Martin & Sunley 2020). Moreover, other critical debates revolve around methods and indicators of how to measure resilience (Martin & Sunley 2020), around whether mainly internal or external factors affect regional resilience (multi‐scalarity) (Bristow & Healy 2020), and around the apparent neoliberal thinking behind regional resilience (MacKinnon & Derickson 2013). Finally, Sweeney *et al*. (2020) recently point at the insufficient distinction made in the literature between regional resilience leading to growth and to decline.

Overall, regional resilience is a highly complex process of different stages, strongly affected by the specific nature, duration and scale of the shock, as well as several impact factors, such as experiences, regional economic structures and state policies at several spatial scales.

## Regional Resilience, Pandemic Crises and COVID‐19

As has been emphasised in the previous section, shocks differ concerning their nature, duration and scale. The current COVID‐19 crisis is a typical example of a pandemic crisis, which can be considered as global shocks, ‘a rapid onset event with severely disruptive consequences covering at least two continents’ (OECD 2011, p. 12). Pandemic crises, such as SARS in 2002/2003, are foremost health crises, but can have related severe negative economic consequences, both at the supply and demand side of the economy (Rubin 2011; OECD 2011). Therefore, they are often both a health crisis, as well as a related social‐economic crisis (Wilson *et al*. 2020). Moreover, ‘Ill informed decisions about the source or severity of an outbreak may lead to ineffective countermeasures that can have significant economic consequences that are difficult to remedy’ (OECD 2011, p. 29).

Economic problems emerge because of several reasons (Bofinger *et al*. 2020; Feld *et al*. 2020; McKibbin & Fernando 2020). First, the disease leads to workforce absenteeism, not only because of those who are ill, but also because of those who have to take care of other ill people or children, because of school and shop closures (Yu & Aviso 2020). Second, the disease and its contagious character often urges governments to react with a lockdown, which is an official order to control the movement of people or vehicles because of the dangerous situation, in order to contain contagion. The related travel and mobility restrictions come at considerable economic costs, because of strongly decreasing or ceasing demand in all mobility‐related industries, such as tourism, hotels, restaurants, airlines, offline retailing, etc. Moreover, import‐dependent manufacturing industries in regions will suffer from disruptions in their supply with components and semi‐finished goods, leading to strong disruptions in global value chains. It is particularly the tourism/hotel industry that suffer more from a pandemic crisis than manufacturing industries, as the latter can pick up production relatively quickly after the crisis,^1^ whereas the tourism industry suffers from long‐term uncertainty and health concerns of citizens. Third, part of the state budget is temporarily shifted to the health sector, which can lead to strongly increasing budget deficits in other sectors. Fourth, economic problems emerge because of interdependencies and the systemic characters of pandemic crises as global crises, leading to cascading and knock on effects to large parts of the economy. Because of the economic shock, investments and output strongly fall, leading to lower demand for goods and services, affecting asset prices negatively which tightens financial conditions, which lead again to falling investments.

Moreover, there are two other typical features of pandemic crises, such as the current COVID‐19 crisis. First, often the disease emerges locally, and then spreads with a time delay, as can be clearly observed during the current COVID‐19 crisis – it first started in Wuhan, China, and later spread to Europe and America, with a high risk of further spreading to many developing countries, in Africa, for example. That means national and regional economies do not suffer at the same time from the health and economic crisis to the same extent, which has complex consequences for global supply chains.

Second, another fundamental and specific issue related to pandemic crises and particularly to the current COVID‐19 is the tension between disease containment policies, on the one hand, and the speed of economic recovery as part of the resilience, on the other hand (see previous section). According to Massaro *et al*. (2018, p. 1) ‘containment interventions intended for a straightforward reduction of the risk may have net negative impact on the system by slowing down the recovery of basic societal functions’. Therefore, in order to assess regional resilience, differences in national and regional containment policies and regulations need to be taken into account (Peckham 2013, 2020; Djalante *et al*. 2020; Hale *et al*. 2020), as they have a potential effect on regional resilience. We can also observe strong differences concerning state financial support toolkits for economic recovery (e.g. the amount of money spent, differentiated support measures), particularly between industrialised and emerging and development countries, which might affect regional resilience (Economist 2020a; OECD 2020).

From an economic‐geographic perspective, the effects of pandemic crises on regional economies and industries, as well as regional resilience, have not been abundantly studied in the past. A short literature review shows that there has only been done work on some regions in East Asia. Examples include a study on the economic effects of SARS on Beijing (Beutels *et al*. 2009), as well as on tourism regions in China (Zeng *et al*. 2005), and the effects of the MERS outbreak (2015–2016) on local and regional authorities in South Korea (Kim *et al*. 2017) and on the tourism industry in South Korea (Joo *et al*. 2019). Concerning the current COVID‐19 crisis, some first studies assess the effects of the crisis on regions, often aiming at giving regional policy‐makers recommendations on how to react to the crisis. Examples include studies assessing the effects of COVID‐19 on all regional economies in Germany (Ehrentraut *et al*. 2020), the Netherlands (Aalders & Raspe 2020) and Italy (ascani *et al*. 2020), as well as on individual regional economies, such as Schleswig‐Holstein in Germany (Schrader *et al*. 2020) and the Basque Country in Spain (Wilson *et al*. 2020).

In the following section, we will analyse the regional economic effects of the COVID‐19 pandemic crisis in China and will give an outlook for other countries in the final section.

## Short‐Term Regional Resilience During the Current COVID‐19 Pandemic Crisis: The Case of China

Although previous regional resilience work commonly analyses regional economic resilience and recovery over a period of years (Davies 2011) or decades (Martin & Sunley 2020), in this research note we will use this term to assess the economic impact of COVID‐19 and the recovery of Chinese regions over a much shorter period (several months). We are of course aware that it would take much longer to tell the complete story of China's regional resilience under the COVID‐19 crisis, but with the data currently available, we can at least give some first indications of long‐term regional resilience in China. Moreover, a careful assessment of the current situation in different regions during this crisis could lead to more targeted and better designed policy suggestions.

### Where is China now in the COVID‐19 shock?

According to Bouey (2020), the outbreak of COVID‐19 in China can be distinguished into three stages. Stage one was characterised by the awakening to the epidemic from December 2019 to January 20, 2020. Stage two, which ran from 21 January 2020 to 21 February 2020, was marked by quarantine and shutdown, and it was in this period that Wuhan became the epicentre of the virus. The third stage, which started on 21 February 2020, is featured by ‘back to work’ within China, while the epidemic is spreading globally to different continents. We are, therefore, currently in a period of some economic recovery in China, while in other parts of the world, the strong influence of the coronavirus continues and heavy containment measures are still being implemented. From a regional resilience perspective, large parts of China are now in the period of ending the temporary ‘resistance’ phase (i.e. reach the bottom) and entering the temporary ‘recovery’ stage, according to Martin *et al*.’s (2016) categorisation of interrelated elements of resilience (Figure 1). Later stages in the process, such as economic re‐orientation and renewal, need to be investigated in the near future with more sophisticated data.

### How hard is China’s economy hit by COVID‐19?

Table 1 shows some key economic indicators on economic performance between December 2019 and February 2020. Nearly all these indicators showed negative growth rates compared to the same period in 2019. Prominently, the cumulative growth rate of industrial value added turned to –13.5 in February, with state‐owned enterprises being influenced to a less extent than private enterprises (Economist 2020b). Moreover, the year‐on‐year growth rate of passenger traffic plummeted to –88.3% with freight volume decreased to –30%. Most strikingly, the Purchasing Managers’ Index (PMI), a key gauge of manufacturing activity fell to 29 per cent in February from 53 per cent in the earlier months.

**Table 1:** Key economic indicators (December 2019‐ February 2020).

	December 2019	January 2020	February 2020
Cumulative growth of industrial value added (%)	5.7	‐	−13.5
Cumulative value‐added growth of state‐owned and state‐holding enterprises (%)	4.8	‐	−7.9
Cumulative value‐added growth of private enterprises (%)	7.7	‐	−20.2
Composite PMI (%)	53.4	53	28.9
Year‐on‐year growth rate of passenger traffic (%)	−3.3	−10.1	−88.3
Year‐on‐year growth rate of freight volume (%)	−17	‐	−29.9
Cumulative growth rate of real estate investment (%)	9.9	‐	−16.3

To understand the potential economic damage, UNCTAD (2020) distinguishes three main channels of disruption: demand, supply and finance. On the demand side, a combination of declining income, shifting sentiment and the absence of a vaccine can be expected to negatively impact private spending. In China, this has been proven by Chinese deposits data published recently by the central bank. RMB deposits increased by 8.07 trillion yuan in the first quarter of this year, an increase of 1.76 trillion yuan year‐on‐year (The People's Bank of China 2020). On the supply side, a sudden stop of factory production in most parts of China has caused disruptions in global supply chains. The concern is that exports of both manufactured final goods and of commodity inputs will begin to weaken in the coming months as the demand from western industrialised economies is estimated to decrease strongly because of the influence of COVID‐19 in such main consumer markets. Finally, concerning finance, the large number of private small‐ and medium‐sized enterprises (SMEs), which accounts for 60 per cent of the economy and 80 per cent of jobs, has felt the impact most. According to a recent survey by the Chinese Association of Small and Medium Enterprises more than 85 per cent of SMEs will run out of cash within three months and will face severe problems in raising funds in the short term (see PWC 2020).

**Figure 2:**
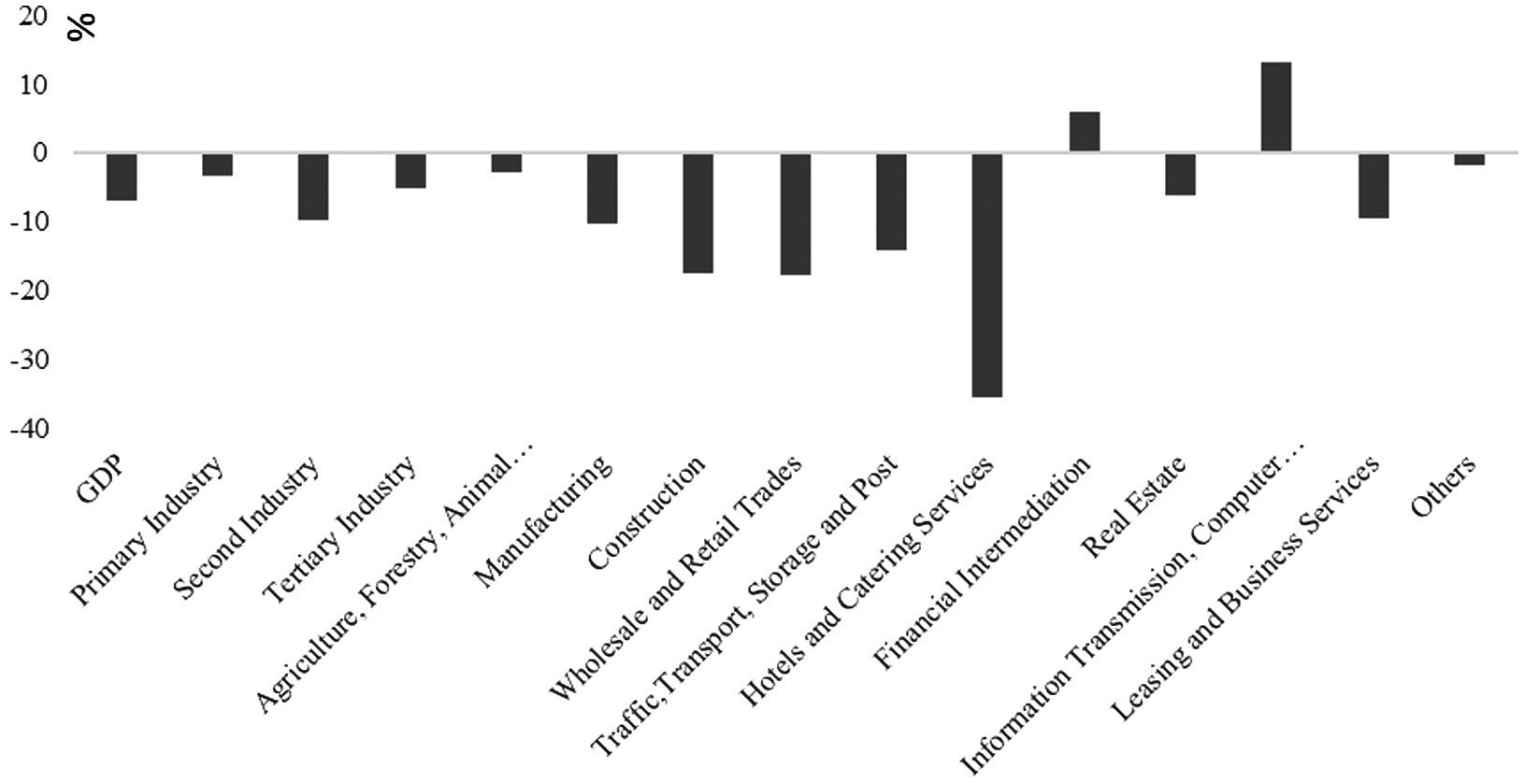
Growth rate of different sectors in the first quarter of 2020. *Source*: own compilation based on data from National Bureau of Statistics of China.

In addition to such macroeconomic indicators, it is essential to look closer at the influence of the crisis on the different economic sectors. As is shown in Figure 2, most economic sectors have been hit by COVID‐19, although the degrees tend to vary. Specifically, the most severely damaged sectors include hotels and catering services (–35.3%), construction (–17.5%), wholesale and retail trade (–17.8%), and traffic and transport (–15%). Given the features of pandemic crises mentioned earlier, as well as the stringent mobility restriction policies that have been adopted by the local and central governments to contain the spread of the disease, it is not surprising that the economic activities in aforementioned sectors were most severely damaged.

**Figure 3:**
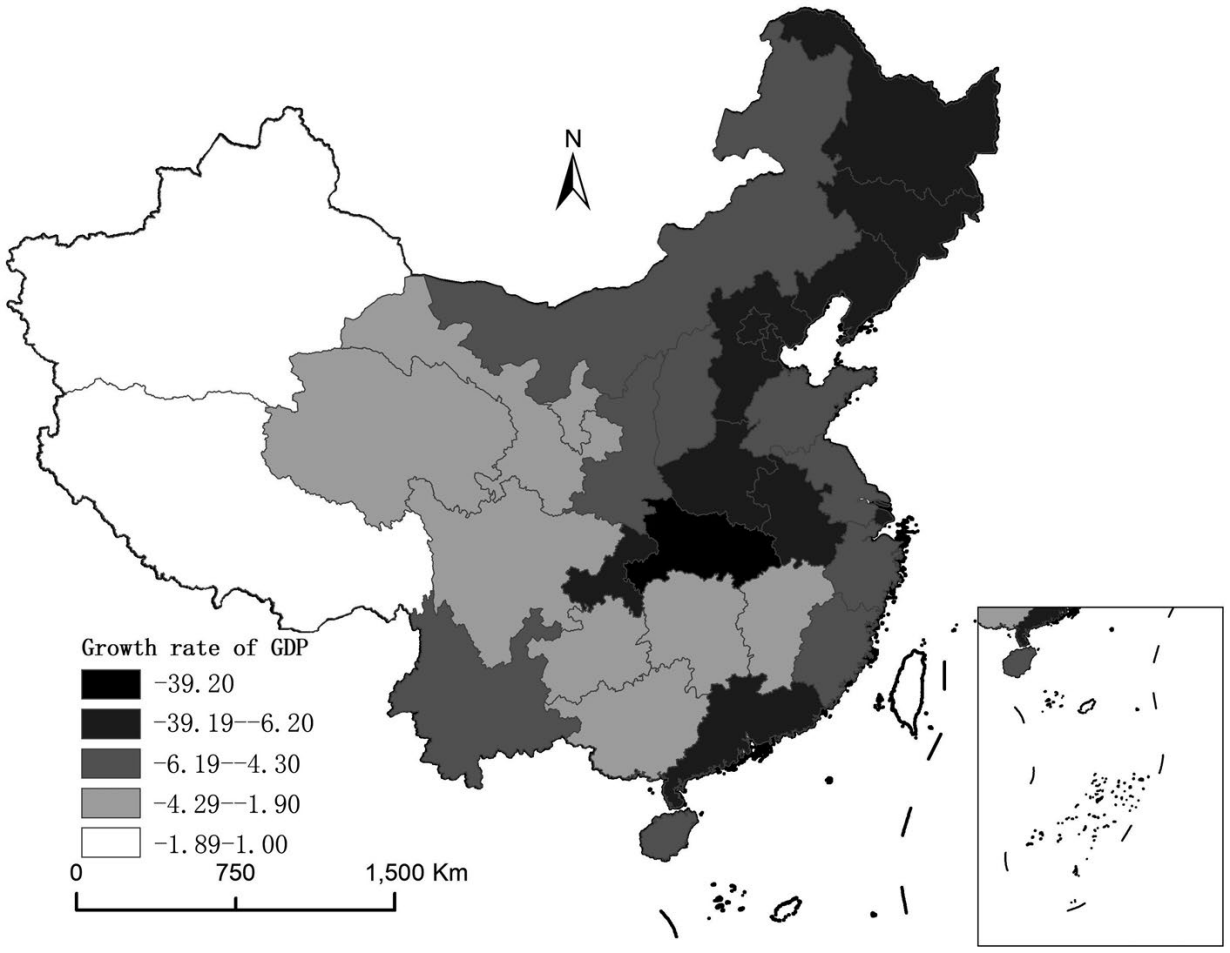
Provincial GDP growth rate in the first quarter of 2020. *Source*: own compilation based on data from National Bureau of Statistics of China.

**Figure 4:**
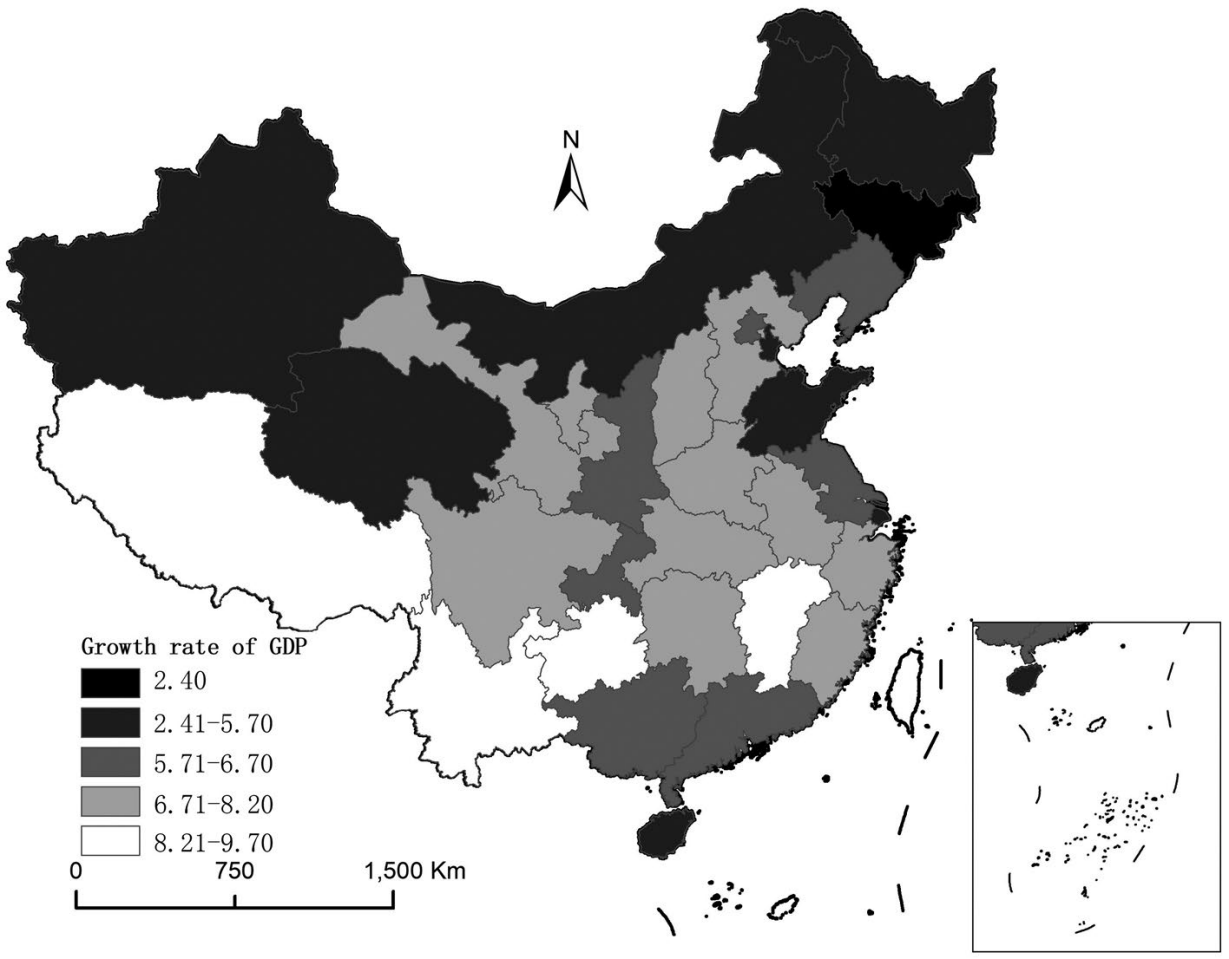
Provincial GDP growth rate in the first quarter of 2019. *Source*: own compilation based on data from National Bureau of Statistics of China.

Looking closer into the different provinces within China, the geographical differences of the economic impact of COVID‐19 can be well observed. According to the recently published first quarter GDP growth rate of Chinese provinces in 2020, almost the whole country has suffered economically from this pandemic crisis, though the degree of influence tends to differ between different provinces (Figures 3 and 4). The epicentre of Hubei province has suffered most strongly and experienced an unprecedented decline of GDP growth rate at –39 per cent. This is exceptional if one considers that the GDP growth rate for the province during the first quarter in 2019 was around 7 per cent. The other strongly hit regions include Hubei’s neighbouring provinces, such as Henan, Anhui, and Chongqing. Although they were not hit by COVID‐19 to the same degree as Hubei, the economic activities in such neighbouring regions have also been severely constrained, due to the implementation of stricter inter‐provincial border control after Wuhan, Hubei, has been identified as the epicentre in China. Overall, eastern provinces were hit much harder economically by COVID‐19, than their counterparts in inland China. This has significant negative outcomes for the overall economic performance of the whole country, as these coastal regions used to be (and still are) the most prosperous regions in China.

### Signs of economic recovery of Chinese regions?

Although the disease has heavily hit the Chinese economy, there are, however, signs of recovery recently. According to the data from the National Bureau of Statistics, the PMI in March has unexpectedly risen to the pre‐crisis level of 53 per cent in March. Although such a single‐month rise does not indicate that the production has returned to pre‐crisis levels, it is often been seen as the first major positive indicator on the business front. In different parts of the country, an increasing number of enterprises have resumed their production in late February and early March. Moreover, although most of the economic indicators still remained negative when compared to the same period in 2019, the situation has improved strongly when compared to the economic indices in February 2020. Taking a short‐term regional resilience perspective, one could potentially argue that the majority of provinces in the country have passed the worst situation (resistance), and hence are entering into the temporary recovery phase (Table 2).

**Table 2:** Key economic indicators (February‐March 2020).

	February 2020	March 2020
Cumulative growth of industrial value added (%)	−13.5	−8.4
Cumulative value‐added growth of state‐owned and state‐holding enterprises (%)	−7.9	−6
Cumulative value‐added growth of private enterprises (%)	−20.2	−11.3
Composite PMI (%)	28.9	53
Year‐on‐year growth rate of passenger traffic (%)	−88.3	−73
Year‐on‐year growth rate of freight volume (%)	−29.9	−13
Cumulative growth rate of real estate investment (%)	−16.3	−7.7

### Potential factors influencing the short‐term economic resilience of Chinese regions: some first estimations

As we mentioned earlier, different provinces in China showed distinct short‐term economic recovery. But why is this the case? In this subsection, we analyse eight factors that might potentially have influenced the short‐term economic recovery/resilience of different provinces. Specifically, we use the first quarter provincial GDP growth rate as an indicator to measure short‐term regional economic resilience. We then use GDP per capita in 2019 as indicator for a region’s overall economic development level. Other factors are also taken into account in our calculation. They include population density, the proportion of added value from secondary and tertiary industries, the proportion of employees in the hotel and catering service sector, foreign trade dependence (total revenue of exports and imports divided by GDP), and the ratio of revenue generated by state‐owned enterprises to revenue from private enterprises. Moreover, given the fact that the severity of the disease might potentially influence regional short‐term resilience, the infection rate per million people is also considered.

Table 3 shows the Pearson correlation between such identified factors and the first quarter GDP growth rates of different provinces. Incorporating Hubei province, the economically most severely damaged region, into the calculation, one could see that most of the indicators chosen have shown negative correlation with the provincial GDP growth rate. It means that all the aforementioned factors had a negative influence on regional short‐term resilience. However, only the infection rate per million people has passed the significance test (significant at 0.01 level), and hence, had a significant and negative correlation with the GDP growth rate. The indicator of ratio of revenue from state‐owned enterprises to revenue from private enterprises has shown a positive, though non‐significant relation with the GDP growth rate. This means that the higher the share of state‐owned enterprises in the local economy, the stronger the local economy has recovered in the short run.

**Table 3:** *The correlation between potential influencing factors and GDP growth rate*.

	Hubei included		Hubei excluded	
	Pearson correlation	Sig. (2‐tailed)	Pearson correlation	Sig. (2‐tailed)
GDP per capita	−0.167	0.369	−0.351	0.057
Population density	−0.099	0.598	−0.373*	0.042
Proportion of added value of secondary industry	−0.067	0.719	0.077	0.687
Proportion of added value of tertiary industry	−0.004	0.983	−0.240	0.202
Proportion of employees in Hotel and Catering Services	−0.035	0.850	−0.211	0.263
Foreign trade dependence	−0.070	0.708	−0.513**	0.004
Ratio of revenue from state‐owned enterprises to revenue from private enterprises	0.131	0.481	0.101	0.594
Infection rate per million people	−0.941***	0.000	−0.404*	0.027

Since Hubei was the epicentre of the COVID‐19 pandemic, and hence showed unique features in comparison to other provinces, in the following Pearson correlation analysis, we will exclude Hubei province, so as to check the correlation of the aforementioned indicators with the GDP growth rate in the rest provinces. As is shown in Table 3, in addition to the indicator of ratio of revenue from state‐owned enterprises to revenue from private enterprises, the proportion of added value of secondary industry also tended to be positively related to the GDP growth rate, although such correlation did not pass the significance test. Moreover, while all the other indicators remain negatively related to the GDP growth rate, three of them have stood out to be significant, including population density, foreign trade dependence, and infection rate per million inhabitants. This indicates that in the short run, the regional resilience of China’ provinces were negatively influenced by the respective regions’ population density, the dependence of regional economy on foreign trade, and the severity of the disease.

### Policy instruments that are important for the long‐term regional resilience in China

In the literature, it is often argued that in and after a global shock such as the current COVID‐19, the role of support policies is often expected to be very important (Martin & Sunley 2020). Since COVID‐19 in China reached its peak in mid‐February, the government has switched its policy to restart the economy. Table 4 summarises some of the recent key measures taken at the national level to help boosting the economy. Specifically, the state‐owned banks tend to play a key role in designing stimulative fiscal and monetary policies to facilitate national economic recovery. The Central Bank, for example, has provided RMB1.2 trillion to ease corporate borrowing. Moreover, it has also adjusted its national interest rate to a relatively low level to encourage the flow of money from banks to the market. On the other hand, the State Council has also decided on different policy portfolios to provide further support to the trapped small businesses. In a recent Executive Session of the State Council, Premier Li Keqiang has sent a strong signal to different departments that the work focus of this year needs to be shifted from guaranteeing a six per cent GDP growth rate to ensure stability in six key aspects of the economy. These include the employment of the population, basic livelihood of the people, the mainstay of the market, food and energy security, the stability of industrial chains and the operation of local authorities.

**Table 4:** Key measures taken by the central government.

Issuing organisations	Content
China’s Central Bank	Monetary stimulus of RMB1.2 trillion in February to ease borrowing costs and funds availability; Cutting the interest rate to 2.5%
China Development Bank	Issuing $2bn bonds in global bond markets
China’s three government‐run policy banks	Lending RMB 350bn to SMEs at preferential rates
Several SOEs	Raising $4bn Covid bonds
State Council	Encouraging private banks to postpone interest payments on loans to SMEs until the end of June; ordering large SOEs to increase lending to SMEs by at least 30% in 2020; ensure stability in six key aspects of the economy

**Figure 5:**
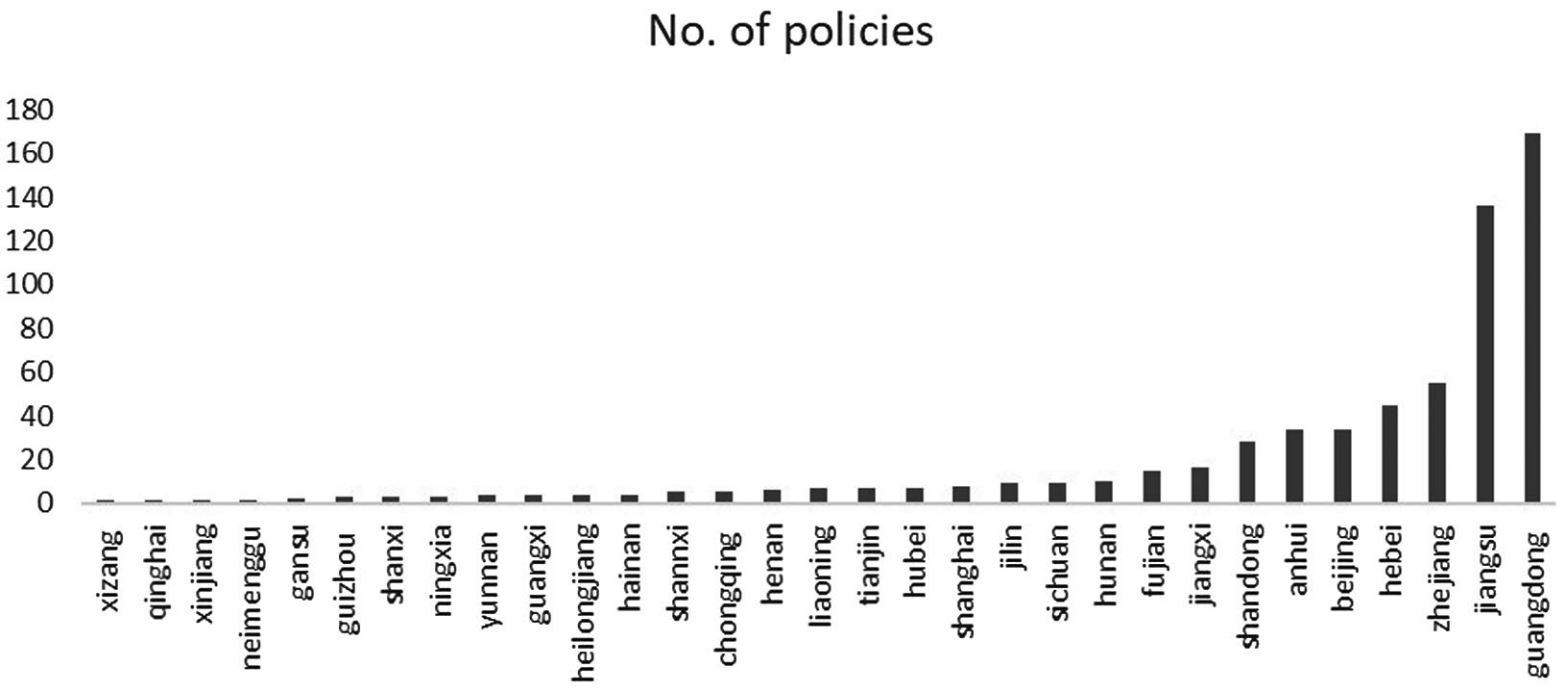
Number of monetary policies related to economic boosting in COVID‐19. *Source*: own compilation based on website of the State Council of China.

In addition to such fiscal, monetary and executive policies, the central government also gave the local governments a great deal of autonomy in implementing and designing new support measures. If one looks at the number of (monetary) policies made by different provinces in the last two months to tackle economic problems caused by COVID‐19 (Figure 5),^2^ it becomes clear that Guangdong, Jiangsu, Zhejiang and Hebei are among the top regions in designing policies in stimulating local economic recovery. Guangdong, the province that has suffered most strongly from the earlier SARS, learnt lessons from previous experience, and was hence quick in revealing the confirmed cases, locking down cities, and more recently in quarantining returned migrants. And it was also among the first provinces that realised the importance of designing effective support measures in facilitating regional economic recovery (Southcn.com 2020). The other provinces with high number of support policies, Jiangsu, Zhejiang and Hebei, are regions with high proportions of private enterprises, which are expected to be mostly affected by the virus (Bouey 2020). Authorities in these regions foresee a strong increase in unemployment and bankruptcies after the crisis, and are hence very active in designing and implementing monetary‐related policies to help private SMEs to weather the current turmoil. We expect such proactive monetary stimulus to be beneficial to the declining regional economies in most parts of China in the forthcoming months and years.

### In every crisis, there is an opportunity: what are the new opportunities that can be identified in the current COVID‐19 crisis?

Martin and Sunley (2020) recently point out the relation between the intensity and duration of the shock and the different kind of transformations that could occur: if the impact of a shock or a crisis is low in intensity and short in duration, regions can be expected to be most likely experiencing the bounce‐back‐like resilience. Whereas if the impact of a shock is high in intensity and long in duration, then, regions are more like to experience the transformative type of resilience, which lead to long‐term structural changes. However, they do not specify to what extent the impact of a shock can be called ‘intensive’, and its duration be seen ‘long’. Of course, it is beyond the purpose of this research note to tease out this issue, but even during the relatively short period of the COVID‐19 crisis in China, we could already spot some promising opportunities emerging from this crisis. Typical examples have been observed in the IT and financial sectors (see Figure 2). Many new business models and technologies (e.g. remote office, online education, digital platform, medical, artificial intelligence, 5G, etc.) have been further developed and applied in this crisis and their influence is expected to be sustained even after it. Therefore, in the long run, regions or cities with a strong base in the aforementioned industries will not only be able to bounce‐back quickly from the pandemic, but more importantly, they are also expected to sustain their competitive advantages and turn their technological advancements into economic benefits (e.g. Shenzhen, Hangzhou, Beijing, Shanghai).

## Preliminary Conclusions and Outlook

In capitalistic systems, regional economic development is an inevitable shock‐prone process (Martin 2018). Despite being criticised, regional resilience is potentially a valuable way of thinking about impacts and implications of shocks and disturbances, such as COVID‐19, for regional economies, and what to do about it and it also has relevance for policy‐making. So far, the bulk of regional resilience literature in economic geography, however, focuses on the effects of the financial crisis 2008 on regional resilience. Little work has been done on regional resilience during and after pandemic crises, which will certainly change in the near future due to the scale of the current COVID‐19 global crisis and recession. This huge amount of work in the near future will doubtlessly feed back into conceptual debates on regional resilience (Martin & Sunley 2020).

Pandemic crises in general and COVID‐19 have particular characteristics. In fact, the situation is rather complex as different kinds of resilience play a role at the same time during this crisis. We can namely observe health resilience (the population recovering from the disease), the resilience of the health system (Legido‐Quigley *et al*. 2020) (which might be related to varieties of capitalism), and economic resilience (recover from the economic consequences) and psychological resilience (again living together without fear for infection). Concerning regional resilience, we can conclude based on a first analysis of Chinese regions, that a complex combination of the characteristics of the current COVID‐19 crisis, the institutional experience of dealing with previous pandemic and epidemic crises, governmental support measures, as well as regional industrial structures, affect the recovery and resilience rates of Chinese regions.

A caveat in the analysis of global pandemic shocks such as the current COVID‐19 is that such a global crisis usually consists of several sub‐shocks (i.e. the immediate economic recession caused by reduced mobility and a further shrinkage caused by declining demand in different parts of the world). These sub‐shocks should be considered as a whole when analysing the impact of the specific crisis. Therefore, a long‐term perspective is necessary in the future to better understand regional resilience in the context of the COVID‐19 shock. However, a closer examination of the current short‐term resilience in different regions within China helps to formulate more localised and targeted policy proposals.

In this research note, we could only present a first analysis of resilience based on a limited number of quantitative data available, measuring short‐term resistance and recovery in China. Further stages in the typical process of regional resilience (Figure 1), that are long‐term in character and focus on adaptability and qualitative changes, need to be investigated in the future, not only in China, but also in all countries affected by COVID‐19. For these studies, we need, in addition to quantitative data, qualitative, place‐specific, contextualised analyses of regional resilience. In comparison to resistance and recovery, which are relatively easy to measure with quantitative data, the more interesting and challenging parts of regional resilience are the reorientation, recoverability and reorganisation stages, as has been shown in the second section. To unpack these parts of the process after COVID‐19 is a future challenge and will need more in‐depth, qualitative research into their mechanisms in a multi‐scalar and comparative way. More research is also needed into the role of transformative agencies (Bristow & Healy 2014; Kurikka & Grillitsch 2020), such as institutional entrepreneurs and place‐based leadership, regional policy intelligence, experiences and lessons from previous experiences in order to fully understand regional resilience after COVID‐19. Moreover, these qualitative analyses will allow us to find out whether regional resilience will be confined to moving back to the normal, pre‐crisis situation (adaptation), or whether shocks are critical junctures and windows of change leading to transformative resilience and hence to new paths emerging out of the crisis.

## Notes

1 If the pandemic is leading to a shrinkage of demand for certain manufactured goods because of the reduction of citizens’ income, then we could of course also expect a strong negative impact of such crisis on the manufacturing sector.

2 We are fully aware that compared to the number of policies designed, the amount of stimulus funds provided by different provinces might be more relevant for regional economic resilience in the long run. However, by reading very carefully all the COVID‐19 related policy documents made by different provincial governments, we realised that not all provinces have provided concrete budgets in their support schemes. Moreover, the stimulus funds also came in different forms (e.g., tax reduction for enterprises, support funding, loans, etc.), hence making it difficult to compare the absolute amount of money.
